# Phenotypic and genotypic characterization of *mcr-1*-positive multidrug-resistant* Escherichia coli* ST93, ST117, ST156, ST10, and ST744 isolated from poultry in Poland

**DOI:** 10.1007/s42770-021-00538-8

**Published:** 2021-06-10

**Authors:** Katarzyna Ćwiek, Anna Woźniak-Biel, Magdalena Karwańska, Magdalena Siedlecka, Christine Lammens, Ana Rita Rebelo, Rene S. Hendriksen, Maciej Kuczkowski, Monika Chmielewska-Władyka, Alina Wieliczko

**Affiliations:** 1 The Faculty of Veterinary Medicine, Department of Epizootiology and Clinic of Birds and Exotic Animals, Wrocław University of Environmental and Life Sciences, Pl. Grunwaldzki 45, 50-366 Wrocław, Poland; 2 University of Antwerp, Campus Drie Eiken Universiteitsplein 1, D.S.624, 2610 Wilrijk, Belgium; 3 WHO Collaborating Center for Antimicrobial Resistance in Foodborne Pathogens and European Union Reference Laboratory for Antimicrobial Resistance, National Food Institute, Technical University of Denmark, Kemitorvet Building 204, 2800 Lyngby, Denmark

**Keywords:** *Escherichia coli*, *Mcr-1*, Multidrug resistance, MLST, Biofilm, Sequence type

## Abstract

**Background:**

A plasmid-mediated mechanism of bacterial resistance to polymyxin is a serious threat to public health worldwide. The present study aimed to determine the occurrence of plasmid-mediated colistin resistance genes and to conduct the molecular characterization of *mcr-*positive *Escherichia coli* strains isolated from Polish poultry**.**

**Methods:**

In this study, 318 *E. coli* strains were characterized by the prevalence of *mcr1–mcr5* genes, antimicrobial susceptibility testing by minimal inhibitory concentration method, the presence of antimicrobial resistance genes was screened by PCR, and the biofilm formation ability was tested using the crystal violet staining method. Genetic relatedness of *mcr-1*-positive *E. coli* strains was evaluated by multilocus sequence typing method.

**Results:**

Among the 318 *E. coli* isolates, 17 (5.35%) harbored the *mcr-1* gene. High antimicrobial resistance rates were observed for ampicillin (100%), tetracycline (88.24%), and chloramphenicol (82.35%). All *mcr-1-*positive *E. coli* strains were multidrug-resistant, and as many as 88.24% of the isolates contained the *bla*_TEM_ gene, tetracycline (*tetA* and *tetB*), and sulfonamide (*sul1, sul2*, and *sul3*) resistance genes. Additionally, 41.18% of multidrug-resistant, *mcr-1*-positive *E. coli* isolates were moderate biofilm producers, while the rest of the strains showed weak biofilm production. Nine different sequence types were identified, and the dominant ST was ST93 (29.41%), followed by ST117 (17.65%), ST156 (11.76%), ST 8979 (11.76%), ST744 (5.88%), and ST10 (5.88%). Moreover, the new ST was identified in this study.

**Conclusions:**

Our results showed a low occurrence of *mcr-1-*positive *E. coli* strains isolated from Polish poultry; however, all the isolated strains were resistant to multiple antimicrobial agents and were able to form biofilms at low or medium level.

## Introduction

Antimicrobial resistance (AMR) has emerged as one of the most important global threats to human health in the last few decades. The increasing resistance of Gram-negative bacteria isolated from poultry is receiving high attention, especially in terms of public health protection, but also in the ability to successfully treat bacterial infections in birds. Resistant bacteria can be transmitted from animals to humans via direct contact between animals and humans, or through the food chain and the environment [1]. A crucial issue seems to be the more frequent isolation of Gram-negative strains resistant to colistin from slaughter animals, e.g., poultry, pigs, and calves [2–4].

In poultry production, colistin (polymyxin E) has been widely administered for the treatment and metaphylaxis of avian colibacillosis, gastroenteritis, and diarrhea to reduce high incidence and mortalities. Such overuse and/or misuse of antibiotics contribute to the development and spread of AMR among poultry strains and flocks, leading to the emergence of multidrug-resistant (MDR) pathogens [5].

The mechanism of action of colistin is the ability of the drug to bind to the surface structures of the bacterial cell membrane (phospholipids and lipopolysaccharides (LPS)), which increases its permeability and weakens the osmotic integrity of the cytoplasmic membrane, while resistance to colistin is an effect of the inefficient binding of polymyxins to the lipid A moiety of LPS due to the 4ʹ-phosphoethanolamine (PEA) modification of lipid A on the LPS [6]. Colistin resistance may be encoded chromosomally or by the *mcr* genes located on mobile genetic elements in plasmid DNA. Chromosomally mediated resistance to colistin is caused by single nucleotide polymorphism in *pmrAB*, *phoPQ*, *mgrB*, and/or *pmrD* genes, resulting in modification of lipid A [7]**.** In 2015, Liu et al. [8] reported the first case of a plasmid-mediated colistin resistance mechanism, designated *mcr-1*, in *E. coli* and *Klebsiella pneumoniae*. Since then, an increasing number of *mcr* genes have been identified. At present, 10 different *mcr* genes and their variants have been described, and these discoveries indicate a rapid evolution of plasmid-mediated colistin resistance gene family [3, 9–15]. The *mcr-1, mcr-2*, and *mcr-3* genes were originally characterized on plasmids in *Enterobacteriaceae*, but have recently been identified on chromosomes in *Moraxella* spp. and *Aeromonas veronii* [16, 17]. Additionally, *mcr-4*, *mcr-5, mcr-6, mcr-7*, and *mcr-8* genes, compared to those listed above, have been described relatively recently. In 2019, the novel *mcr-*9 homolog was detected in the clinical isolate of *Salmonella* Typhimurium in the USA [18]. The first case of identification of the latest variant *mcr-10* in an *Enterobacter roggenkamp* strain was reported in 2020 [19]. Currently, *mcr* genes have been globally distributed, and an in silico analysis showed their presence on plasmids and their high prevalence among *Enterobacteriaceae* strains isolated from humans, animals, food, and environment [15]. Moreover, this resistance could be easily transferred to other bacterial cells during cell division or horizontal gene transfer (e.g., conjugation or transduction) [20, 21].

It is worth noting that the transferable plasmid-mediated genes that could rapidly spread between bacterial species and hosts, and the possible transmission of resistance genes due to cross-contamination between food-production chains, animals, and humans have raised worldwide concern in recent years [6, 8, 22].

In human medicine, polymyxins are used only for the emergence of MDR bacteria, which are responsible for severe infections and deaths, as a last resort antimicrobial agent against these “super bacteria.” The spread of diverse antimicrobial resistance genes in *Enterobacteriaceae*, e.g., colistin and quinolone resistance genes, is well known among the bacteria within this family [23, 24].

Currently, with the increase in resistance of bacteria to commonly used antimicrobial agents, polymyxins are also used as the last resort therapy for biofilm-related infections. Biofilm is a multicellular structure, which is defined as a community of cooperating bacteria that adhere to biological or nonbiological surfaces contained in the extracellular polymeric matrix [25]. Bacteria embedded in the inner layers of the biofilm may show less susceptibility to antibiotics due to increased horizontal gene transmission, modification of the antibiotic target or cell permeability, and the use of efflux pumps or the expression of hydrolyzing enzymes. Colistin can act against metabolically inactive bacterial cells in the inner layers of the biofilm. Because of this property, colistin is the subject of research in which combined antibiotic therapy is recommended as a treatment for biofilm-related infections caused by Gram-negative bacteria [26, 27].

In the last few years, several reports have been published on the detection and characterization of colistin-resistant *E. coli* strains isolated from slaughter animals [4, 8, 28–31]. Studies on colistin resistance and the prevalence of resistance-associated genes among bacterial strains from various sources have been conducted worldwide. In Poland, studies on colistin-resistant *E. coli* strains isolated from slaughter and wild animals were conducted by Wasyl et al. [32, 33], Zając et al. [34], and Majewski et al. [35]. Those studies were mainly focused on the antimicrobial resistance of *E. coli* strains, the presence of *mcr* genes, and molecular identification and characterization of resistance mechanisms. However, there is still scarce research on the relationship between AMR, genotypic characterization (AMR genes, multilocus sequence typing — MLST), and the ability of biofilm formation in *mcr-1-*positive *E. coli* strains isolated from poultry in Poland.

The present study aimed to assess the prevalence of the *mcr* genes among *E. coli* strains isolated from different types of poultry (broilers, laying hens, turkeys, geese, and ducks), to determine the antimicrobial susceptibility phenotypes of *mcr-*positive strains, and to evaluate the association of observed phenotypes with the presence of AMR genes, MLST sequence types, and the ability of biofilm production by these strains.

## Material and methods

### Isolate collection

A total of 318 *E. coli* isolates were obtained from the AGRO-VET Veterinary Laboratory in Wrocław, Poland. The strains were collected during 2016–2020 from different types of poultry: broilers (n = 161), turkeys (n = 72), breeder broilers (n = 37), laying hens (n = 20), ducks (n = 14), and geese (n = 14). Strains were isolated from organs with lesions or from cloacal swabs and identified using standard microbial and chromogenic media for coliform bacteria, especially those for selective isolation of *E. coli.*

### DNA isolation

Total DNA of all 318 *E. coli* strains was isolated from 18- to 20-h culture of the strains in LB medium (BIOCORP, Warszawa, Poland) incubated at 37 °C. For DNA extraction, the commercial Genomic Mini® kit (A&A Biotechnology, Gdynia, Poland) was used and the procedure was performed according to the manufacturer’s protocol. The purity and concentration of the obtained DNA were assessed using a spectrophotometer, and the amount of DNA was estimated to be approximately 30 ng/µl. As good quality DNA, the A260/A280 ratio of 1.7–2.0 and A260/A230 greater than 1.5 were taken [36]. The obtained DNA of strains was protected and stored at − 20 °C until further tests.

### PCR-based screening of colistin resistance genes

Multiplex PCR was used to amplify part of the five colistin resistance genes *mcr-1*, *mcr-2*, *mcr-3*, *mcr-4*, and *mcr-5* in all 318 *E. coli* strains according to published protocol [37] with minor modification. Each PCR reaction was performed in 25 µl total volume consisting of 2.5 µl 10 × DreamTaq Green Buffer (Thermo Fisher Scientific, Waltham, USA), each primer at 0.2 µM final concentration (Genomed, Warszawa, Poland), 0.2 mM nucleotide mix (Thermo Fisher Scientific), 1 U of DreamTaq Green Polymerase (Thermo Fisher Scientific), and 1 µl DNA template. The thermal profile included initial denaturation at 95 °C for 10 min, followed by 30 cycles of denaturation at 95 °C for 30 s, annealing at 63 °C for 90 s, elongation at 72 °C for 60 s, and final elongation at 72 °C for 10 min. The PCR products were run on 1.5% agarose gel with Midori Green DNA Stain (Nippon Genetics Europe GmbH, Dueren, Germany) at 100 V. PCR products with the expected base pair size (320, 715, 929, 1116, and 1644 bp, respectively) were subsequently sequenced and then analyzed using BioEdit (v. 7.2.5) software and GenBank database to confirm the test results.

As positive controls for *mcr* genes (*mcr-1*, *mcr-2*, *mcr-3*, *mcr-4*, *mcr-5*) detection, the following strains were used in this study: *E. coli* KP81 and *E. coli* KP37 for the detection of *mcr-1* and *mcr-2* genes, respectively. Strains were given by Christine Lammens from the University of Antwerp in Belgium. Moreover, *E. coli* SQ352, *E. coli* DH5α, and *Salmonella paratyphi* B-SA01718 were used as positive controls for *mcr-3*, *mcr-4*, and *mcr-5* gene detection, respectively. These strains were obtained from the European Union Reference Laboratory for Antimicrobial Resistance at the National Food Institute, Technical University in Denmark.

### Antimicrobial susceptibility test

The examination of antimicrobial susceptibility to selected antimicrobial agents was performed by determination of minimal inhibitory concentration (MIC) using the commercial system MIC Sensititre EU Surveillance *Salmonella*/*E. coli* EUVSEC Plate (Thermo Fisher Scientific, WalthamAZI, USA) according to the manufacturer’s instructions. Resistance breakpoints to fourteen antimicrobial agents, namely gentamicin (GEN), ampicillin (AMP), cefotaxime (CTX), ceftazidime (CAZ), meropenem (MEM), nalidixic acid (NAL), ciprofloxacin (CIP), chloramphenicol (CHL), azithromycin (AZM), colistin (CST), tetracycline (TET), tigecycline (TGC), sulfamethoxazole (SMX), and trimethoprim (TMP), were determined for *mcr-1-*positive strains. The tested *E. coli* strains were classified as susceptible (S) or resistant (R) based on EUCAST guidelines, version 10.0, 2020 [38]. In the case of absence of limit values for selected antimicrobials, the guidelines of the Clinical Laboratory Standards Institute (CLSI) were used to analyze the results [39]. The reference strain of *E. coli* ATCC 25,922 was used as test control. In addition, the investigated isolates were categorized as multidrug resistant (MDR), when they were simultaneously resistant to at least three antimicrobial agents from different classes of antimicrobial agents [30].

### Detection of antimicrobial resistance genes

PCR amplification of the genes related to resistance to beta-lactams (*bla*_CTX-M_, *bla*_SHV_, and *bla*_TEM_), quinolones (*qnrA*, *qnrB*, *qnrC*, *qnrD*, *qnrS*, *qepA*, and *aac(6′)-Ib-cr*), phenicols (*cat1*, *cat2*, and *cat3*), tetracyclines (*tetA*, *tetB*, *tetC*, and *tetD*), and sulfonamides (*sul1*, *sul2*, and *sul3*) was performed for the *E. coli* isolates carrying the *mcr-1* gene [30, 31, 40]. These genes were chosen as a molecular resistance mechanism of *E. coli* to the selected antimicrobials used for antimicrobial susceptibility tests in this study. Sequenced PCR products of resistance genes, collected during previous studies, were used as positive controls in this study [41].

### Biofilm formation by mcr-1-positive E. coli strains

Biofilm formation was tested in 96-well flat polystyrene microtiter plates (Corning Inc., New York, USA), based on a modified protocol previously described [26, 42]. The *mcr-1-*positive *E. coli* strains were cultured overnight in LB medium, adjusted to a density of 0.5 McFarland units, and then diluted in a proportion of 1:9 with fresh LB medium. A volume of 200-µl aliquots of each dilution was then dispensed into a microtiter plate well, and each bacterial suspension was inoculated in 6 wells. Negative controls for the test were uninoculated LB medium. To compare biofilm formation, the results of *mcr-1-*positive *E. coli* isolates, *E. coli* ATCC 25,922, and three collected *E. coli* strains without *mcr-1* were used. The microtiter plate was incubated for 24 h in aerobic conditions at 37 °C without shaking.

After incubation, the supernatant was discarded, and the microtiter plate was washed three times gently with 250 µl of phosphate buffer solution (PBS). Microplates were then stained with 200 µl of 0.1% crystal violet for 15 min, washed thrice with PBS, and dried for 30 min. Adherent cells were solubilized with 200 µl of 95% ethanol. OD_590_ (optical density) was measured using an automated microplate reader Spark (Tecan Group Ltd., Männedorf, Switzerland). Based on the OD produced by bacterial biofilms and established cut-off value (ODc), the strains were classified into the following categories: no biofilm former — OD ≤ ODc, weak biofilm former — ODc < OD ≤ 2ODc, moderate biofilm former — 2ODc < OD ≤ 4ODc, or strong biofilm former — 4ODc < OD [25, 43].

### MLST of the mcr-1-positive strains

The *mcr-1*-positive *E. coli* isolates were analyzed using the MLST method to determine the sequence types (STs). Depending on the alleles of seven basic metabolism genes (housekeeping genes), namely *adk*, *fumC*, *gyrB*, *icd*, *mdh*, *purA*, and *recA*, the Achtman MLST method was performed using the EnteroBase (v.1.1.2) database (http://enterobase.warwick.ac.uk), and the protocol described by Wirth et al. [44]. Sequences of seven housekeeping genes were concatenated for each isolate using BioEdit (v. 7.2.5), and then the phylogenetic tree was reconstructed by the neighbor-joining method with 1000 bootstrap trials, and Kimura’s correction using MEGA 6.0 software [45–47].

## Results

### PCR-based screening of colistin resistance genes

Among all the tested *E. coli* strains (n = 318), 17 isolates (5.35%) were *mcr-1* positive, whose presence was confirmed by the 100% of nucleotide identity of the amplicons when they were sequenced (https://blast.ncbi.nlm.nih.gov/Blast.cgi). The *mcr-2*, *mcr-3*, *mcr-4*, and *mcr-5* genes were not detected in any of the collected *E. coli* strains. Most of the *mcr-1* harboring *E. coli* strains were isolated from turkeys (9; 52.94%), followed by seven strains (41.18%) from broilers and one strain (5.88%) from goose.

### Antimicrobial susceptibility

**Fig. 1:**
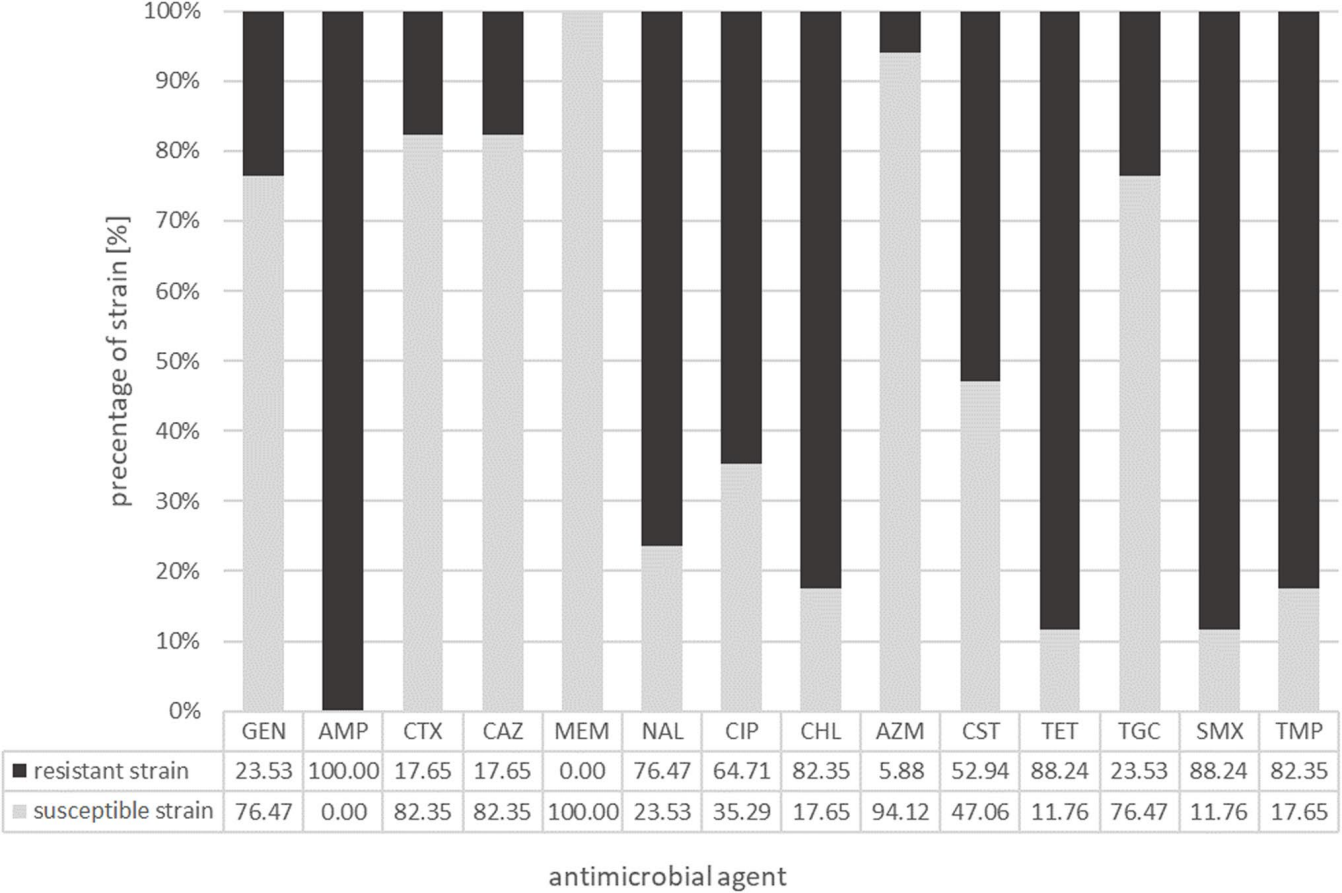
Antimicrobial susceptibility of the mcr-1 harboring *E. coli* strains (n = 17). GEN, gentamicin; AMP, ampicillin; CTX, cefotaxime; CAZ, ceftazidime; MEM, meropenem; NAL, nalidixic acid; CIP, ciprofloxacin; CHL, chloramphenicol; AZM, azithromycin; CST, colistin; TET, tetracycline; TGC, tigecycline; SMX, sulfamethoxazole; TMP, trimethoprim

Antimicrobial susceptibility of the *E. coli* strains carrying the detected *mcr-1* gene is shown in Table 2 Occurrence of resistance genes to antimicrobial agents, resistance profiles, biofilm formation, and sequence types (STs) in mcr-1-positive Escherichia coli strains (n = 17)[Display Image Removed]White square — lack of resistance gene; black square — presence of resistance gene.GEN, gentamicin; AMP, ampicillin; CTX, cefotaxime; CAZ, ceftazidime; MEM, meropenem; NAL, nalidixic acid; CIP, ciprofloxacin; CHL, chloramphenicol, AZM, azithromycin; CST, colistin; TET, tetracycline; TGC, tigecycline; SMX, sulfamethoxazole; TMP, trimethoprim.Biofilm formation: +  +  + — strong biofilm former, +  + — moderate biofilm former, + — weak biofilm former. Table 1 and in Fig. 1. All *mcr-1-*positive *E. coli* strains (n = 17) were susceptible (100%) to meropenem (MIC value 0.03 µg/ml). In addition, most strains were susceptible to azithromycin (94.12%, MIC range of 2–8 µg/ml), ceftazidime (82.35%, MIC range of 0.5–1 µg/ml), cefotaxime (82.35%, MIC range of 0.25–1 µg/ml), and tigecycline (76.47%, MIC range of 0.25–0.5 µg/ml). Similarly, the percentage of strains susceptible to gentamicin was relatively high (76.47%; MIC range of 0.5–1 µg/ml).

**Table 1:** Minimal inhibitory concentration (MIC) (μg/ml) of *mcr-1*-positive *Escherichia coli* strains (n = 17)

Origin	Strain	MIC value (µg/ml)													
		GEN	AMP	CTX	CAZ	MEM	NAL	CIP	CHL	AZM	CST	TET	TGC	SMX	TMP
Turkeys	RF1/17	*0.5*	** > 64**	**4**	**4**	< *0.03*	*4*	**8**	*8*	*8*	*1*	** > 64**	*0.5*	*8*	**8**
	RF2/17	*1*	** > 64**	**4**	**4**	< *0.03*	*4*	*0.015*	*8*	*8*	*1*	** > 64**	*0.5*	** > 1024**	**32**
	RP1/17	*1*	** > 64**	*0.25*	*0.5*	< *0.03*	** > 128**	** > 8**	**32**	*2*	*1*	** > 64**	*0.25*	** > 1024**	** > 32**
	RP2/17	*1*	** > 64**	*0.25*	*0.5*	< *0.03*	** > 128**	** > 8**	**32**	*2*	*1*	** > 64**	*0.25*	** > 1024**	** > 32**
	RP3/17	*0.5*	** > 64**	*0.25*	*0.5*	< *0.03*	** > 128**	*0.25*	** > 128**	*8*	*1*	** > 64**	*0.5*	*8*	*0.25*
	AE02/18	*1*	** > 64**	< *0.25*	< *0.5*	< *0.03*	** > 128**	**8**	< *8*	*4*	**8**	** > 64**	**1**	** > 1024**	< *0.25*
	AE21/18	** > 32**	** > 64**	< *0.25*	< *0.5*	< *0.03*	< *4*	*0.03*	**64**	< *2*	*2*	*4*	**2**	** > 1024**	*1*
	AE178/20	< *0.5*	** > 64**	< *0.25*	< *0.5*	< *0.03*	** > 128**	**8**	**128**	**16**	< *1*	** > 64**	< *0.25*	** > 1024**	** > 32**
	AE342/20	**32**	** > 64**	< *0.25*	< *0.5*	< *0.03*	< *4*	< *0.015*	**32**	< *2*	**4**	**64**	< *0.25*	** > 1024**	** > 32**
Broilers	RW1/17	*0.5*	** > 64**	*0.5*	*0.5*	< *0.03*	** > 128**	*0.25*	**64**	*2*	**8**	*2*	*0.5*	** > 1024**	** > 32**
	RW2/17	** > 32**	** > 64**	*1*	*0.5*	< *0.03*	** > 128**	** > 8**	**128**	*8*	**8**	** > 64**	*0.5*	** > 1024**	** > 32**
	RW3/17	*8*	** > 64**	** > 4**	**4**	< *0.03*	** > 128**	** > 8**	**128**	< *2*	*2*	** > 64**	< *0.25*	** > 1024**	** > 32**
	AE64/18	** > 32**	** > 64**	< *0.25*	*1*	< *0.03*	** > 128**	** > 8**	** > 128**	*8*	**8**	** > 64**	**2**	** > 1024**	** > 32**
	AE71/18	*1*	** > 64**	< *0.25*	< *0.5*	< *0.03*	** > 128**	*0.25*	**64**	*4*	**8**	**64**	< *0.25*	** > 1024**	** > 32**
	AE256/20	< *0.5*	** > 64**	< *0.25*	< *0.5*	< *0.03*	** > 128**	**4**	**128**	< *2*	**8**	** > 64**	< *0.25*	** > 1024**	** > 32**
	AE257/20	< *0.5*	** > 64**	< *0.25*	< *0.5*	< *0.03*	** > 128**	**4**	**128**	< *2*	**8**	** > 64**	< *0.25*	** > 1024**	** > 32**
Geese	AE05/18	*1*	** > 64**	< *0.25*	< *0.5*	< *0.03*	** > 128**	**8**	**128**	*4*	**8**	** > 64**	**1**	** > 1024**	** > 32**

Bold — resistance to an antimicrobial agent; italics — susceptible to an antimicrobial agent.

*GEN*, gentamicin; *AMP*, ampicillin; *CTX*, cefotaxime; *CAZ*, ceftazidime; *MEM*, meropenem; *NAL*, nalidixic acid; *CIP*, ciprofloxacin; *CHL*, chloramphenicol; *AZM*, azithromycin; *CST*, colistin; *TET*, tetracycline; *TGC*, tigecycline; *SMX*, sulfamethoxazole; *TMP*, trimethoprim.

On the other hand, all strains (100%) were resistant to ampicillin (MIC > 64 µg/ml). The occurrence of resistant isolates to tetracycline (88.24%), chloramphenicol (82.35%) nalidixic acid (76.47%), and ciprofloxacin (64.71%) was also high. MIC values ​for tetracycline-resistant strains (n = 15) were in the range of 64 µg/ml (2 strains) and > 64 µg/ml (13 strains). The ranges of MIC values ​for chloramphenicol-resistant strains were varied and were as follows: 32 µg/ml (n = 3), 64 µg/ml (n = 3), 128 µg/ml (n = 8), and > 128 µg/ml (n = 2). Among the *E. coli* strains resistant to nalidixic acid, the MIC value for all strains was > 128 µg/ml, while the MIC value for ciprofloxacin ranged between 4 and ≥ 8 µg/ml.

It is worth emphasizing that among the 17 *mcr-1-*positive *E. coli* isolates, only nine strains (52.94%) were resistant to colistin; among these strains, the MIC value was 8 µg/ml for eight strains (88.89%). In all *E. coli* strains resistant to sulfamethoxazole (15; 88.24%), the MIC value exceeded 1024 µg/ml, while in strains resistant to trimethoprim (14; 82.35%), the MIC value ranged from 8 to ≥ 32 µg/ml.

Interestingly, among the 17 *mcr-1*-positive *E. coli* strains isolated from different poultry types, the resistance to the 13 tested antimicrobial agents was similar. An exception was the resistance to colistin, wherein six of strains were derived from broilers (35.29%), two from turkeys (11.76%), and one *E. coli* isolate (5.88%) from goose.

On the basis of the interpretation of MIC breakpoint values, according to EUCAST recommendations [38], 15 resistance profiles of the isolated *E. coli* strains were described. All the investigated isolates were MDR. The 15 resistance profiles obtained in this study are presented in Table 2.

The most common resistance profile consisted of six classes of antimicrobial agents: AMP + NAL + CIP + CHL + CST + TET + SMX + TMP and was observed in two strains of *E. coli* (AE256/20 and AE257/20) isolated from the internal organs of broilers. Another two *E. coli* strains isolated from the organs of turkey (RP1/17 and RP2/17) had a resistance profile of five classes of antimicrobial agents: AMP + NAL + CIP + CHL + TET + SMX + TMP; their resistance profiles (even for 7 classes of antimicrobial agents) were noted in single strains of *E. coli* isolated from different sources and places (turkeys, broilers, and geese; organs and cloacal swabs).

It is worth noting that among the *mcr-1*-positive *E. coli* strains isolated from chicken broilers, two strains (11.76%) exhibited resistance profile to as many as 7 classes of antimicrobial agents, four strains (23.53%) to 6 classes, and one strain (5.88%) to 5 classes of antimicrobial agents. In contrast, among isolates from turkeys, most strains showed a resistance profile to 5 classes of antimicrobial agents (5 strains, 29.41%). The remaining *E. coli* isolates showed resistance profiles to 6 classes (2 strains; 11.76%) and 4 classes (2 strains, 11.76%) of antimicrobial agents. The isolate from the goose was resistant to antimicrobial agents from 6 classes.

### Occurrence of resistance genes and biofilm formation

The occurrence of the selected resistance genes among the *mcr-1-*positive *E. coli* strains isolated from poultry is shown in Table 2. The results showed the frequent presence of one of the beta-lactam resistance genes (*bla*_TEM_) (88.24%) in the isolated *E. coli* strains, with the simultaneous absence of the other two resistance genes to this class of antibiotics (*bla*_SHV_ and *bla*_CTX-M_). The percentage of strains that harbored the sulfonamide resistance genes was as follows: *sul1 —* 70.59%, *sul2 —* 70.59%, and *sul3 —* 52.94%. All the three genes were detected in 29.41% of *mcr-1-*positive *E. coli* strains. In addition, 70.59% of *E. coli* isolates showed the presence of the *tetA* gene and 35.29% had the *tetB* gene. None of the investigated isolates harbored the *tetC* or *tetD* gene. The phenicol resistance gene *cat1* was detected only in five isolates (29.41%), and it was one of the three resistance genes tested for this class of antimicrobial agents. In contrast, among the seven fluoroquinolone resistance genes tested (*qnrA*, *qnrB*, *qnrC*, *qnrD*, *qnrS*, *qepA*, and *aac(6′)-Ib-cr*), only one *E. coli* strain (5.88%) isolated from turkeys had *aac(6′)-Ib-cr.* No differences were observed in the presence or absence of the selected resistance genes depending on the source of *E. coli*.

**Table 2:**
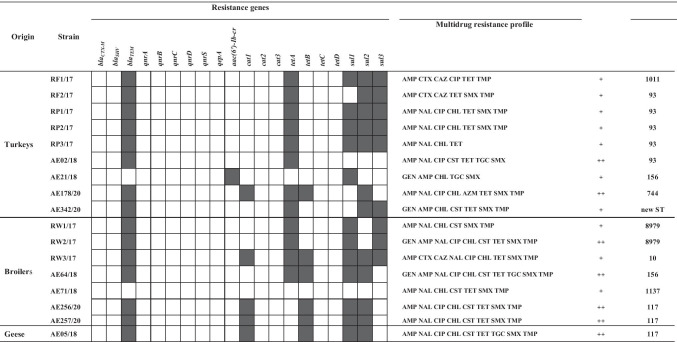
Occurrence of resistance genes to antimicrobial agents, resistance profiles, biofilm formation, and sequence types (STs) in *mcr-1*-positive *Escherichia coli* strains (n = 17)

White square — lack of resistance gene; black square — presence of resistance gene.

*GEN*, gentamicin; *AMP*, ampicillin; *CTX*, cefotaxime; *CAZ*, ceftazidime; *MEM*, meropenem; *NAL*, nalidixic acid; *CIP*, ciprofloxacin; *CHL*, chloramphenicol, *AZM*, azithromycin; *CST*, colistin; *TET*, tetracycline; *TGC*, tigecycline; *SMX*, sulfamethoxazole; *TMP*, trimethoprim.

Biofilm formation: +  +  + — strong biofilm former, +  + — moderate biofilm former, + — weak biofilm former.

The results of biofilm assay are presented in Table 2. The results showed that all the 17 *E. coli* strains isolated from poultry origin produced biofilms, although at different levels of intensity. Under the assessed incubation conditions, most strains isolated from turkeys (41.18%) produced biofilm weakly, while the remaining strains (11.76%) were found to be moderate biofilm formers. In contrast, 23.53% of broiler isolates were medium biofilm producers and 17.65% isolated were weak biofilm producers. The only goose *E. coli* strain showed moderate biofilm production. It should be noted that none of the tested strains showed a strong biofilm formation.

#### MLST

**Fig. 2:**
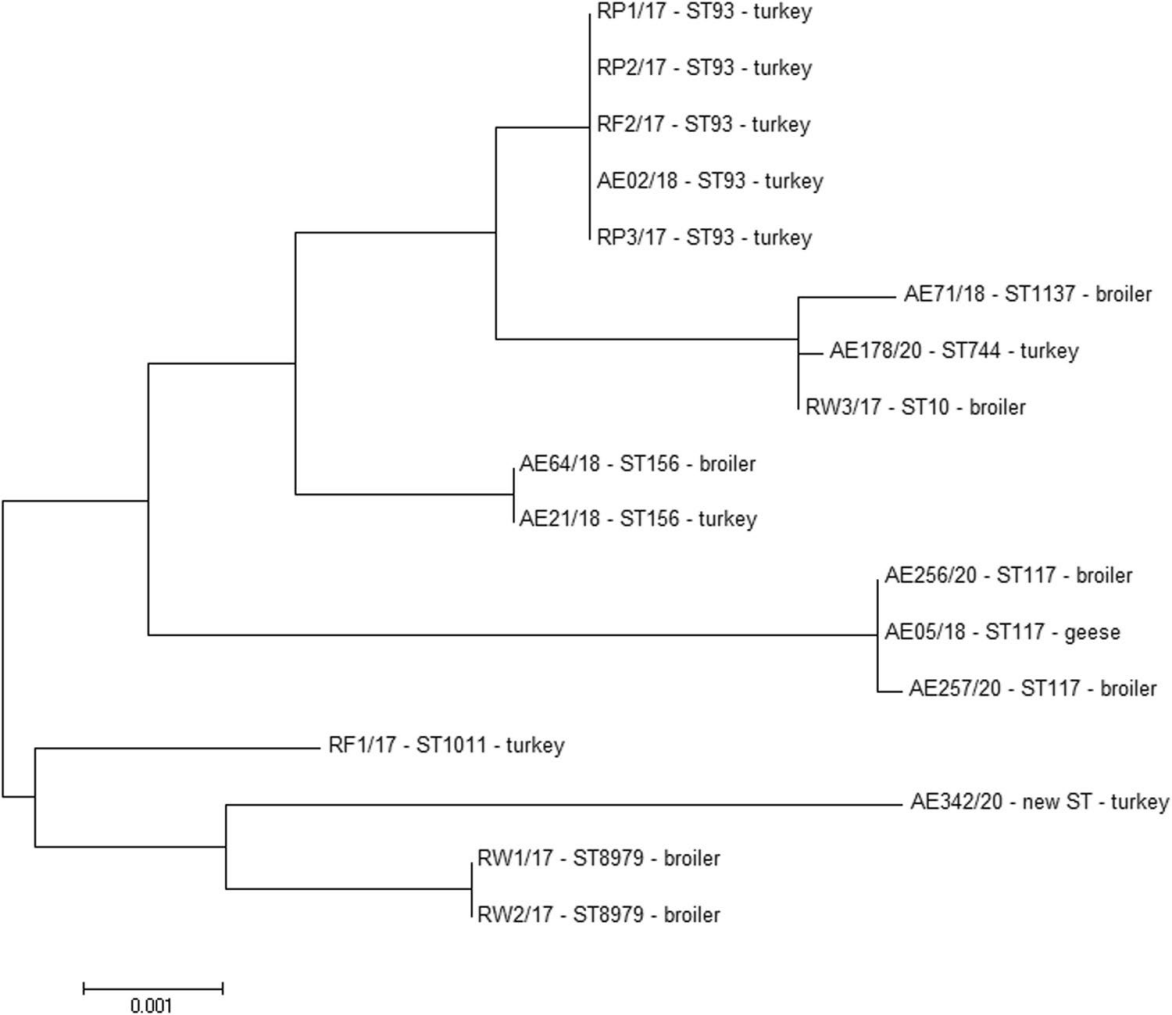
Sequence types (STs) and MLST-based phylogenetic tree of *mcr-1-*positive *E. coli* isolates. The phylogenetic tree was constructed using the neighbor-joining method in MEGA 6.0 software

On the basis of the combination of allelic profiles of the tested housekeeping genes, all 17 *E. coli* strains were assigned to the ST (Fig. 2). All loci showed four or more alleles among the 17 tested strains. The *adk* allele showed the least genetic variability (4 different alleles), whereas *gyrB* was the most genetically diverse (8 different alleles) among all investigated housekeeping genes.

**Fig. 3:**
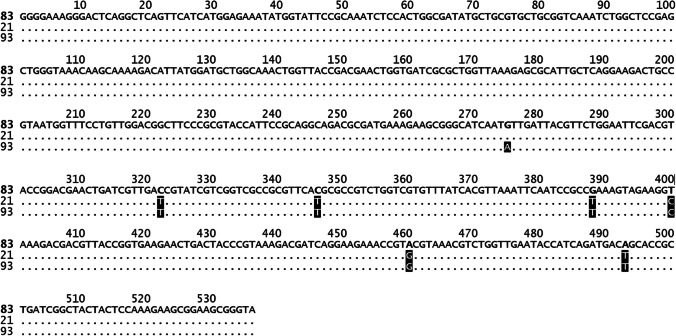
The nucleotide differences in *adk* allele between the new ST and the ST69 clonal complex (ST69 and ST408)

The MLST analysis showed the occurrence of nine STs, among which the most frequent were ST93 and ST117 (five and three isolates, respectively). The ST93 was observed in turkey isolates, while the ST117 in isolates obtained from broilers and from one goose. Moreover, the new ST was identified, which was related to the ST69 clonal complex. This complex includes the ST69 and ST408, which show the difference in *adk* allele (21 and 93, respectively). The new ST has an *adk* 83 allele, which was different in 6 nucleotides in comparison to *adk* 21 allele, and in 7 nucleotides in comparison to *adk* 93 allele. The details of the differences between *adk* alleles are presented in Fig. 3.

The phylogenetic relationship showed that the strains of the same sequence types, such as ST93, ST117, ST156, and ST8979, despite their various origins, were assigned to the same clusters, and had the closest relationship with each other. Moreover, the closely related strains exhibited very similar profiles of carried resistance genes and the level of biofilm formation. All ST93 strains were isolated from turkeys and 60% (3/5) of them showed the same resistance gene profile and weak ability to form biofilms. All of the ST117 strains included isolates that harbored the *bla*_TEM_, *cat1*, *tetB*, *sul1*, and *sul2* genes, and they were moderate biofilm producers.

## Discussion

Colistin resistance genes are widespread worldwide and have been found in *Enterobacteriaceae* from humans, food animals, and food, and among these resistance genes, the *mcr-1* gene is the most frequently isolated one [48, 49]. In Poland, the first *E. coli* strain with the *mcr-1* gene was isolated in 2015 from a 50-year-old patient with a urinary tract infection. The man had contact with farm animals, which may confirm the involvement of animals in the transmission of colistin-resistant strains [50]. Zhang et al. [51] revealed the prevalence of colistin resistance genes (*mcr-1*, *mcr-2*, and *mcr-3*) in various species of poultry, with the highest prevalence of the *mcr-1 gene*, which was obtained from 71.7% of geese, 34.6% of ducks, and 31.8% of broilers. Moreover, a serious concern was the presence of all three *mcr* genes in three separate *E. coli* isolates from broilers.

Our study showed a low occurrence (5.35%) of *mcr-1-*harboring *E. coli* strains isolated from poultry. The other *mcr* genes (*mcr2*–*mcr5*) were not detected in the all analyzed strains; this finding is similar to the results obtained by other authors [52–54]. According to the research conducted in China by Zhao et al. [55], the percentage of *mcr-1-*positive *E. coli* strains isolated from poultry was 15.3%, while it was only 0.34% in Japan [3]. Frequent use of antimicrobial agents in livestock production may lead to higher rate of resistant strain isolation, and commensal bacteria might serve as an indicator of antimicrobial usage for veterinary purposes [56]. A significantly higher percentage of colistin-resistant *E. coli* strains in Poland was confirmed by studies conducted in 2017–2018, where the presence of the *mcr-1* gene occurring in the normal microbiota of chicken broilers, both treated and untreated with colistin sulfate, was tested. Isolates containing the *mcr-1* gene were obtained in 11.27% of strains from untreated flocks and in 19.54% of isolates obtained from flocks treated with colistin [35]. In Europe, the prevalence of *E. coli* strains isolated from poultry and carrying the *mcr-1* gene ranged from 1.5% in the Netherlands [28] to 13.95% in Portugal [54].

Irrgang et al. [57] showed that the prevalence of the *mcr-1* gene depended on the type of poultry production. The highest prevalence of the *mcr-1* gene was detected in turkeys (11.8%), followed by broilers (6.7%), and only three *E. coli* strains were *mcr-1* positive in laying hens (3/1, 809 investigated isolates). In our present study, the proportions remained similar, i.e., the *mcr-1* gene was most frequently present in turkeys (9/72; 12.50%), followed by broilers (7/161; 4.35%). Clemente et al. [54] revealed that as many as 27% of turkey strains and only 2% of the investigated broiler strains harbored the *mcr-1* gene.

In the present study, the highest resistance profile was observed for ampicillin (100%), followed by sulfamethoxazole and tetracycline (88.24% for both), and trimethoprim and chloramphenicol (82.35% *ex aequo*); this finding is in accordance with other studies conducted in Brazil by Crecencio et al. [58] and in Portugal by Manageiro et al. [59]. Manageiro et al. revealed that a high percentage of *E. coli* strains isolated from broilers and turkeys were resistant to the following antimicrobial agents: ciprofloxacin (90.6% and 79.5%), nalidixic acid (88.6% and 73.5%), ampicillin (75.7% and 80%), sulfamethoxazole (69.3% and 71.9%), tetracycline (66.3% and 85.9%), trimethoprim (54.5% and 49.7%), and chloramphenicol (34.2% and 52.4%). Interestingly, multidrug resistance was observed in 81.3% of the isolates. Crecencio et al. [58] showed the highest resistance of retail chicken meat to beta-lactams (39.5%), followed by sulfonamide combined with trimethoprim (36.9%) and polymyxin (33.4%).

In our present study, regarding the obtained MIC values for the selected antimicrobial agents, all the investigated *mcr-1-*positive *E. coli* strains showed MDR profile (resistance to at least three antimicrobial classes). Similar results were obtained by other authors from Brazil, China, Argentina, and Poland [29–31, 35, 60]. Monte et al. [29] showed that most of the *mcr-1-*positive *E. coli* isolates exhibited an MDR phenotype and carried genes conferring resistance to aminoglycosides, quinolones, sulfonamides, and tetracyclines. In the present study as well as in the study of Haenni et al. [61], the association between *mcr* and other resistance elements such as beta-lactamases and the coexistence of the *mcr-1* gene with sulfamethoxazole or tetracycline-resistance genes was observed. In our present study, the most frequently found resistance genes in the *mcr-1-*positive *E. coli* strains were as follows: *bla*_TEM_ (resistance to beta-lactams), *tetA* and *tetB* (resistance to tetracyclines), and *sul1*, *sul2*, and *sul3* genes (resistance to sulfonamides). These findings are in agreement with the results obtained by Zhao et al. [55] who showed that among *mcr-1-*positive *E. coli* strains, the *bla*_TEM_ gene was the most prevalent (100%). β-lactamase encoding genes are usually localized on plasmids that facilitate their spread among Gram-negative bacilli via conjugation. Moreover, β-lactamase encoding plasmids often carry genes conferring resistance to other than β-lactam classes of antibiotics, limiting significantly the therapeutic options [62, 63]. It should be emphasized here that *E. coli* is an opportunistic pathogen that is capable of causing illness in animals and humans; therefore, the isolation of MDR bacteria from food animals is a worldwide public health problem because of potential transfer of resistant pathogens to humans [56] and the possibility of transmission of antimicrobial resistance genes among gut bacteria.

Resistance to fluoroquinolones is either a chromosomally mediated mechanism causing mutation in the quinolone resistance-determining region (QRDR) within the subunits constituting topoisomerases II (GyrA and GyrB) and IV (ParC and ParE) or is encoded by plasmid-mediated quinolone resistance genes (PMQR) [*qnrA*, *qnrB*, *qnrC*, *qnrD*, *qnrS*, *qepA*, *oqxAB*, and *aac(6′)-Ib-cr*], where the *qepA* and *oqxAB* genes encode an efflux pump that decreases intracellular drug levels [23, 64, 65]. Moreover, decreased accumulation of fluoroquinolones because of impermeability of the membrane and/or overexpression of the efflux pump systems has also been established [66]. In the present study, 76.47% and 64.71% of the *mcr-1-*positive *E. coli* strains were resistant to nalidixic acid and ciprofloxacin, respectively, while the presence of the *aac(6′)-Ib-cr* gene was detected in only one strain. This difference between phenotypic resistance to quinolone antibiotics and the presence of the resistance genes is probably due to other resistance mechanisms, listed above, which were not investigated in the present study.

Biofilm formation by *E. coli* strains is one of their mechanisms of virulence and is important in the development of antibiotic resistance. In the present study, most of the investigated strains were weak biofilm producers (58.82%), but a medium biofilm formation ability was observed in strains with multidrug resistance to 5, 6, or 7 classes of antimicrobial agents (41.18%). Also Pavlickova et al. [56] showed the correlation between the prevalence of antibiotic resistance and biofilm formation ability. Moreover, 71% of *E. coli* strains isolated from chicken exhibited weak and medium biofilm production ability, which is in agreement with the results of our present study. In comparison, Crecencio et al. [58] showed that as many as 70.44% of *E. coli* strains isolated from retail chicken meat had moderate to strong biofilm formation ability. These discrepancies may depend on strain properties, culture conditions, environmental factors, and methodology [67]. Regarding the poultry species, medium biofilm-producing *E. coli* strains were most frequently isolated from broilers (23.53%). This observation showed that on the one hand, multidrug resistance of these strains may enhance their virulence, especially in broiler isolates, and on the other hand, the general capacity of the *mcr-1-*positive *E. coli* strains to produce biofilms was at the medium and low level (no strong biofilm producers were observed in this study). Similar results were obtained by Barilli et al. [68], wherein *E. coli* strains isolated from retail meat products (including poultry) were weak biofilm producers. Although the tested in vitro strains did not show a strong biofilm production, it is worth noting that under appropriate in vivo conditions, with insufficient production hygiene, biofilm production may be more effective. Biofilm formation potential appears as an important virulence factor in ensuring the low penetration of antibiotics or disinfectants, and may lead to ineffective treatment.

In the present study, the MLST analysis revealed nine different *E. coli* STs. The most dominant sequence type was ST93 (29.41%), followed by ST117 (17.65%). Our study showed that eight obtained STs (ST93, ST1137, ST744, ST10, ST156, ST117, ST1011, and ST8979) have been isolated and previously identified among poultry in the USA, Europe, Asia, and Australia (data taken from EnteroBase). These STs were also noted in Poland, except for ST117 and ST1011, which were isolated for the first time from poultry source in our study. The newly identified ST was the most related to the ST69 clonal complex, which includes the ST69, widely distributed in the environment, and the ST408 which has been isolated from bovine in the USA.

It is worth noting that in the present study, two strains isolated from broilers were described as ST8979; according to the EnteroBase, this ST has been isolated only twice from the environment in the USA. The ST1137 was deposited in the EnteroBase in 12 cases, including three strains each isolated from poultry in the USA, France, and Kenya. Additionally, three of STs (ST10, ST93, and ST744), obtained in the present study, were detected in *E. coli* from samples of raw poultry meat and liver, which came from Poland and were retailed in the Czech Republic. This investigation suggests that it poses a significant threat to public health [69].

Zhao et al. [38, 55] showed that among the *mcr-1-*positive *E. coli* strains isolated from poultry farms in China, the dominant ST was ST93 (18.62%); this finding is in agreement with the results obtained in the present study. The ST93, ST117, and ST156 were also described in *E. coli* strains obtained from chicken broilers in Egypt [70]. In Switzerland, Zurfluh et al. [71] characterized the ST156 in an *mcr-1-*positive *E. coli* strain isolated from chicken meat, while Hassen et al. [72] revealed the presence of ST117 in a beta-lactamase-producing *mcr-1-*encoding *E. coli* strain isolated from chicken meat samples in Tunisia. The other STs, namely ST10 and ST117 were found in *E. coli* isolated from broilers at slaughter [73] and broiler breeders [74], respectively. In Poland, Zając et al. [34] described 49 STs among the *mcr-1-*positive *E. coli* strains isolated from chicken and turkeys, among which the most common types were ST354 and ST359, which were not observed in our present study. The common STs for both classes of strains, published by Zając et al. [34] and in the present study, were ST10, ST93, ST117, and ST1011. In addition, in the present study, 60.0% (3/5) of ST93 isolates carried the *bla*_TEM_, *tetA*, *sul1*, *sul2, and sul3* genes, and they showed 100% similarity on the MLST phylogenetic tree; this finding may indicate clonal types of these strains.

## Conclusion

In summary, the conducted research confirmed that poultry can be considered as an important reservoir of MDR *E. coli* isolates. A wide range of phenotypic resistance to both antibiotics used in veterinary and human medicine was identified and resistance to colistin, tetracycline, quinolones, and β-lactams was observed among the analyzed strains. Additionally, strains with multidrug resistance to 5, 6, or 7 classes of antimicrobial agents were medium biofilm producers. The co-resistance of plasmid-mediated colistin resistance encoded by *mcr-1* gene and *tet (A* and *B)* or *bla*_TEM_ genes was 88.24%, equally. Furthermore, our findings suggest the diversity in resistance determinants, which could be responsible for the high resistance profiles found in this study. This may pose a threat to public health due to the existing risk of spread of resistance genes among bacterial strains, including their potential ability to transfer antimicrobial resistance to humans.

Due to the fact that Poland is a significant poultry producer in Europe, research on this aspect should be widely conducted in Poland and constantly improved. It is essential to monitor colistin-resistant *E. coli* strains for understanding the prevalence of colistin resistance genes in both human and veterinary medicine, including poultry production.
